# Early trajectories of benthic coral reef communities following the 2015/16 coral bleaching event at remote Aldabra Atoll, Seychelles

**DOI:** 10.1038/s41598-020-74077-x

**Published:** 2020-10-12

**Authors:** Anna Koester, Valentina Migani, Nancy Bunbury, Amanda Ford, Cheryl Sanchez, Christian Wild

**Affiliations:** 1 Marine Ecology Department, Faculty of Biology and Chemistry, University of Bremen, Leobener Straße 6, 28359 Bremen, Germany; 2 Institute for Ecology, Faculty of Biology and Chemistry, University of Bremen, Leobener Straße 5, 28359 Bremen, Germany; 3 Seychelles Islands Foundation, PO Box 853, Victoria, Mahé Seychelles; 4 Centre for Ecology and Conservation, University of Exeter, Cornwall Campus, Penryn, TR10 9FE UK; 5 School of Marine Studies, Faculty of Science, Technology and Environment, University of the South Pacific, Suva, Fiji

**Keywords:** Tropical ecology, Marine biology, Ecology, Ecology

## Abstract

Documenting post-bleaching trajectories of coral reef communities is crucial to understand their resilience to climate change. We investigated reef community changes following the 2015/16 bleaching event at Aldabra Atoll, where direct human impact is minimal. We combined benthic data collected pre- (2014) and post-bleaching (2016–2019) at 12 sites across three locations (lagoon, 2 m depth; seaward west and east, 5 and 15 m depth) with water temperature measurements. While seaward reefs experienced relative hard coral reductions of 51–62%, lagoonal coral loss was lower (− 34%), probably due to three-fold higher daily water temperature variability there. Between 2016 and 2019, hard coral cover did not change on deep reefs which remained dominated by turf algae and *Halimeda*, but absolute cover on shallow reefs increased annually by 1.3% (east), 2.3% (west) and 3.0% (lagoon), reaching, respectively, 54%, 68% and 93% of the pre-bleaching cover in 2019. Full recovery at the shallow seaward locations may take at least five more years, but remains uncertain for the deeper reefs. The expected increase in frequency and severity of coral bleaching events is likely to make even rapid recovery as observed in Aldabra’s lagoon too slow to prevent long-term reef degradation, even at remote sites.

## Introduction

Climate change-induced coral bleaching events are increasing in frequency and severity, threatening the persistence of coral reef ecosystems worldwide. Global warming reduced the time frames between bleaching events from 27 years in the early 1980s to 5.9 years in 2016^1^ and recovery windows are predicted to shorten even further as severe bleaching events are expected to occur annually on 90% of the world’s coral reefs by 2055^2^. In this context, assessing post-disturbance reef trajectories is crucial to understand which conditions favour reef recovery^3,4^.

The metric most widely used to assess reef recovery is the return time of coral cover to pre-disturbance values, hereafter ‘coral recovery’^5,6^. Coral recovery rates are influenced by various environmental and physical factors (e.g. water depth^7,8^, light intensity^7^, nutrient levels^9^, water flow^10^, temperature variability^11,12^). Furthermore, as bleaching susceptibility and rebound potential of corals varies on multiple levels (e.g. among^13,14^ and within taxa^15,16^, across coral growth forms^17^, among symbiont type^18,19^), coral recovery is typically faster than the return to pre-disturbance coral community composition, hereafter ‘reassembly’^6^. Reassembly is a critical aspect of reef recovery, ensuring that the coral community’s traits and functions are restored^6^. Speed of coral recovery and reassembly varies substantially among reefs^6,20,21^ and regions^5^, underlining the complex nature of reef recovery processes.

Reef recovery relies on the growth and propagation of surviving colonies and coral recruitment^3,22,23^ which is influenced by abiotic and biotic conditions and anthropogenic disturbance. High wave exposure, for example, limits coral growth and coral larvae settlement^24^, while coral recruitment and survival can be enhanced by herbivores that control algal turf and fleshy macroalgae and promote crustose coralline algae (CCA) growth^25–27^. These natural drivers of recovery may be disrupted by direct human stressors such as overfishing of herbivores and/or nutrient enrichment, favouring algal proliferation and impeding or preventing reef recovery. This implies that, despite substantial variation in coral recovery and reassembly, both can be promoted through targeted management of direct anthropogenic disturbance^28^.

Consequently, coral reefs removed from direct human stressors serve as a baseline to assess the natural recovery potential in the face of the exacerbating effects of climate change and diminishing time frames for reef recovery. Understanding the variation in bleaching impact and recovery trajectories at such sites can provide crucial information for regional marine spatial planning and climate policies^29^. Aldabra Atoll in the Western Indian Ocean (WIO) offers the opportunity to examine bleaching impacts and subsequent reef trajectory dynamics under minimal direct human disturbance^30,31^. Designated as a Special Reserve, the highest level of national protection, under Seychelles’ legislation in 1981, and inscribed as a UNESCO World Heritage Site in 1982, Aldabra’s marine ecosystem has been protected from commercial fishing pressure for almost 40 years, and human-driven nutrient inputs are absent. Nevertheless, coral bleaching events have caused high coral mortality at Aldabra in 1998/1999 (38% and 66% on the seaward reef at 10 m and 20 m water depth, respectively^32^) and in 2015/2016 (35% in the lagoon at 2 m water depth; 54% at 5 m and 55% at 15 m water depth on the seaward reef^33^).

Here we utilise a unique 5-year data set that covers the aftermath of a major global coral bleaching event at a remote reef system with minimal local human impact. Following the findings of Cerutti et al.^33^ that bleaching induced coral mortality was lower at Aldabra’s lagoon than at the seaward reef after the 2015/16 coral bleaching (35% vs. 55% loss), we examine early post-bleaching reef trajectories at Aldabra Atoll in the context of spatial variations in bleaching impact by: (1) assessing changes of benthic communities across locations between 2014 (pre-bleaching) and 2016 (post-bleaching) and quantifying daily water temperature variability to explore whether this links to spatial differences in bleaching impact^12^, and (2) evaluating the post-bleaching trajectories (recovery/stability/degradation) of the benthic communities at these locations between 2016 and 2019. We use our results to outline expected future prospects for Aldabra’s reefs and the implications for remote reefs elsewhere.

## Results

### Benthic community change directly after bleaching

**Figure 1:**
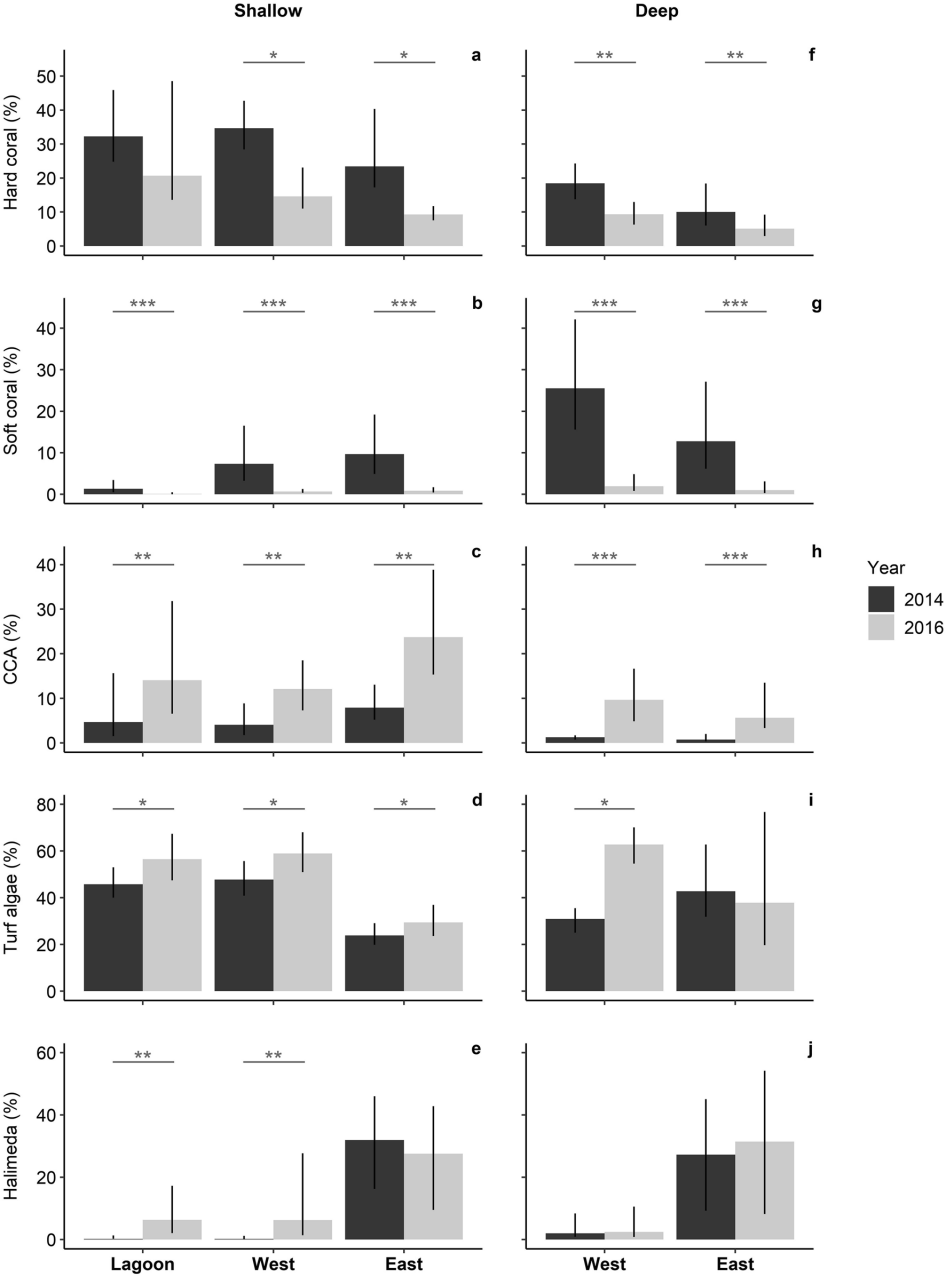
Mean cover of major benthic groups at shallow (**a**–**e**) and deep locations (**f**–**j**) between 2014 and 2016 at Aldabra (transect sections: lagoon n = 6, west n = 10 per depth, east n = 8 per depth). Bars represent back-transformed estimates of GEE analysis with 95% confidence interval. Significant differences across years are indicated with asterisks (*p < 0.5; **p < 0.01; ***p < 0.001).

**Figure 2:**
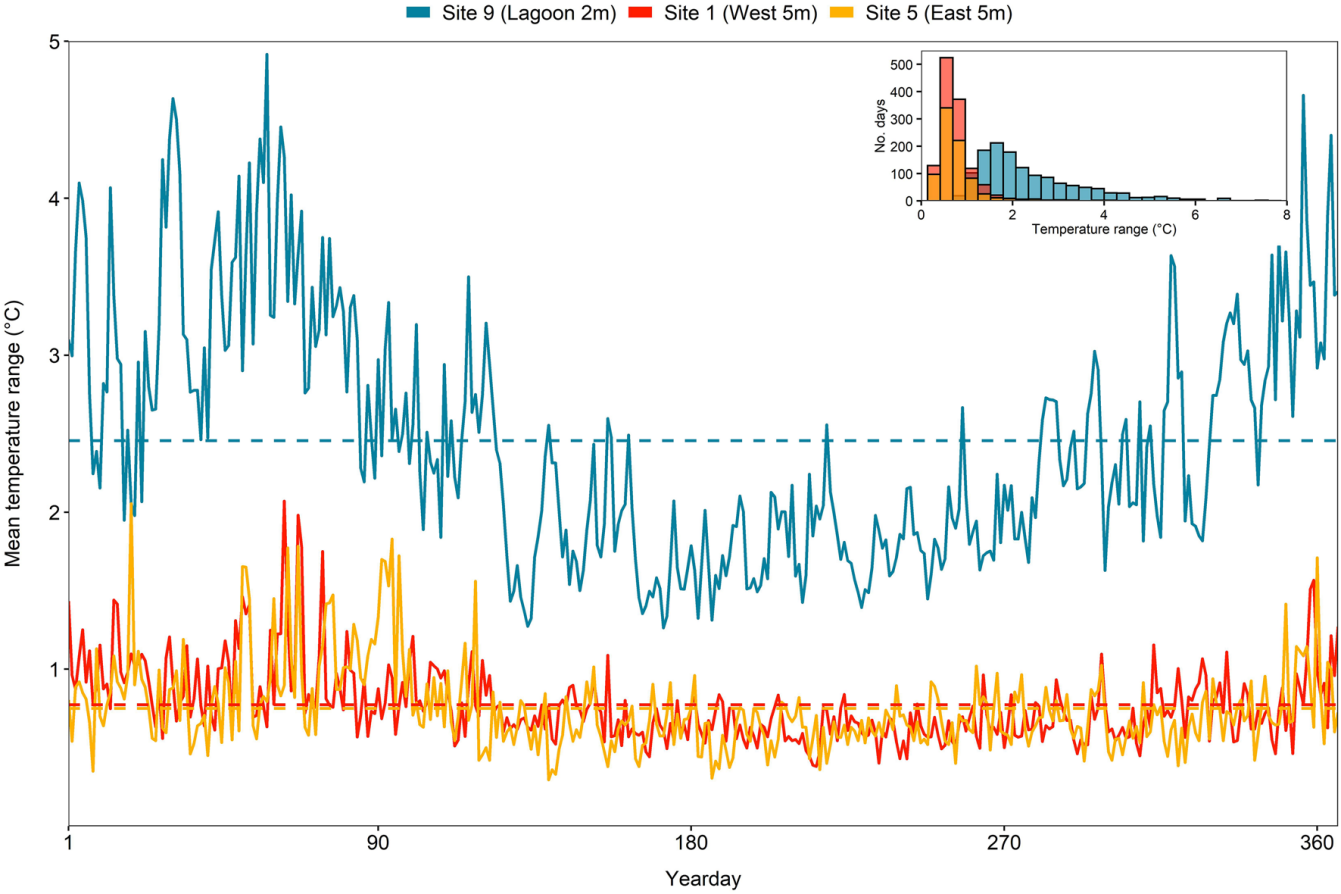
Mean in-situ water temperature ranges (°C) at three representative sites in the lagoon and at the seaward east and west of Aldabra. Solid lines indicate daily mean ranges calculated from temperature records obtained in 30-min intervals between February 2015 and November 2018 (see “Methods”). Dashed lines represent mean daily temperature range within the study period. Inset shows the same data as histogram, representing the number of days during which a given temperature range was recorded at each site.

Overall, between Dec 2014 and Dec 2016, Aldabra’s reefs experienced 53% and 92% reductions of hard and soft corals, respectively. Hard coral reduction, however, was only significant on the seaward reefs, where losses were substantially higher than inside the lagoon (lagoon: − 34%, west shallow: − 56%; east shallow: − 62%, west and east deep; − 51%; Fig. 1a, Supplementary Table S1). In the lagoon, mean daily water temperature range was more than three times higher than the ranges recorded at the shallow seaward reefs (Table 1, Fig. 2). Soft coral cover declined by 91–92% at all locations (Fig. 1b,g), but absolute losses were considerably lower inside the lagoon as soft coral cover there was already < 2% in 2014 (compared to 7–26% at the seaward reefs). At the lagoon and west, CCA and turf algae increased (CCA, lagoon: 5–14%, shallow west: 4–12%, deep west: 1–9%; turf algae, lagoon: 46–57%, shallow west: 48–59%, deep west: 30–62%), together comprising 70% of the benthos at these locations in 2016 (Fig. 1c–i). While calcareous green alga *Halimeda* only increased at the shallow west and the lagoon (from ca. 1 to 6% cover), it remained most abundant at the east (25–29% cover in 2016) and together with CCA and turf algae (i.e. all algae groups combined) comprised 70–81% of the benthos there in 2016 (Fig. 1c–j).

**Table 1:** Differences in mean daily water temperatures (mean, mean maximum, mean minimum) and daily mean temperature variability (range, CV: coefficient of variation of mean) of three shallow reef sites in the lagoon and the seaward west and east of Aldabra during Feb 2015–Nov 2018 (see also Supplementary Figure S2).

Site	Daily temperature (° C)						t test								
	Site 9 (Lagoon)		Site 1 (West)		Site 5 (East)		Site 9 vs. Site 1			Site 1 vs. Site 5			Site 9 vs. Site 5		
Measure	Mean	SD	Mean	SD	Mean	SD	dF	t	*p*	dF	t	p	dF	t	*p*
Mean	27.47	1.71	27.13	1.66	27.24	1.79	729.4	2.7	** < 0.05**	728.7	0.8	> 0.05	726.2	1.8	> 0.05
Max	28.77	1.96	27.45	1.78	27.59	1.82	723.7	9.5	** < 0.001**	729.7	1.0	> 0.05	726.1	8.5	** < 0.001**
Min	26.26	1.34	26.67	1.66	26.83	1.70	706.7	3.7	** < 0.001**	729.5	1.2	> 0.05	700.1	5.0	** < 0.001**
Range	2.45	0.77	0.77	0.25	0.75	0.27	436.8	39.4	** < 0.001**	719.0	1.2	> 0.05	456.0	39.4	** < 0.001**
CV	3.44	1.11	1.82	0.69	1.76	0.75	609.1	23.7	** < 0.001**	725.1	1.2	> 0.05	639.0	24.0	** < 0.001**

Cover of all coral taxa declined significantly between 2014 and 2016 (except for ‘other hard corals’ and *Isopora palifera*, the latter of which slightly increased at the shallow east during 2014–2016) but absolute losses varied across locations (Supplementary Table S2, Supplementary Fig. S1). *Acropora* and *Montipora* were most affected at the shallow west and east, dropping from 2 to 5% cover in 2014 to ≤ 0.7% cover in 2016, a relative reduction of 84–99%. Although relative losses of 83–95% were also recorded for both taxa at the deep locations and the lagoon, absolute losses there were lower, as cover of these taxa was already low in 2014 (deep locations: 0.4–1.7%; lagoon: 0.6–0.9%). Branching *Porites* experienced a relative decline of 83–99% at the seaward reefs (1–4% cover in 2014; < 0.1% cover in 2016), but retained 50% of pre-bleaching cover at the lagoon (6% in 2014, 3% in 2016). Of all taxa, the highest absolute losses were recorded for *Rhytisma* at the seaward west where it reduced from 8 and 26% cover (shallow and deep, respectively) in 2014 to < 0.1% cover at both depths in 2016.

### Post-bleaching trajectories

**Figure 3:**
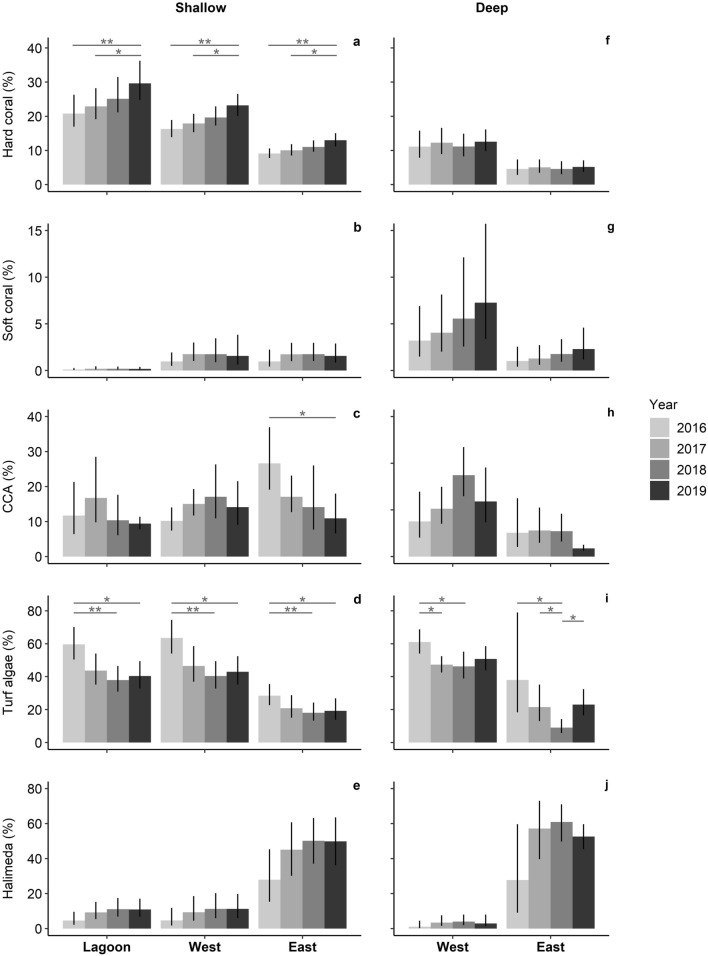
Mean cover of major benthic groups at shallow (**a**–**e**) and deep locations (**f**–**j**) between 2016 and 2019 at Aldabra (transect sections: lagoon n = 9, west n = 15 per depth, east n = 12 per depth). Bars represent back-transformed estimates of GEE analysis with 95% confidence interval. Significant differences across years are indicated with asterisks (*p < 0.5; **p < 0.01).

Except for soft coral cover, which did not change at any location between 2016 and 2019, trajectories of benthic groups varied across locations (Fig. 3, Supplementary Table S3). By 2019, mean hard coral cover had increased at all shallow locations to 13%, 23% and 30% at the east, west and lagoon, respectively (Fig. 3a), equating to 54% (east), 68% (west) and 93% (lagoon) of the pre-bleaching hard coral cover. The absolute annual rate of change in hard coral cover was 1.3%, 2.3% and 3.0% at the east, west and lagoon, respectively (Table 2). From 2019, the projected time until hard coral cover has fully recovered to pre-bleaching levels (2014) is 0.7, 4.8 and 8.5 years at the lagoon, west and east, respectively (Table 2). These are conservative estimates as hard coral cover gains until 2019 were non-linear and accelerated over time (e.g. 1.6–2.0 times higher from 2018 to 2019, than from 2017 to 2018, and 2016 to 2017). Simultaneously with hard coral cover increase, turf algae reduced to below pre-bleaching levels by 2018 (east: 28–18%, west: 64–40%, lagoon: 60–38%; Fig. 3d). However, while benthic communities at the shallow west and lagoon were no longer turf algae-dominated by 2018, and CCA cover remained unchanged during 2016–2019, CCA cover dropped at the shallow east during 2016–2019 (27–11%), with turf algae and *Halimeda* covering 18–21% and 45–50% of the shallow eastern benthos in 2017, 2018 and 2019, respectively (Fig. 3c–e).

**Table 2:** Absolute percentage increase in mean hard coral cover at Aldabra’s shallow locations between 2016 and 2019 and projected time remaining from 2019 for full hard coral recovery (values obtained from back-transformed estimates of GEE analysis).

Location	Absolute hard coral cover increase (%)				Annual rate of change	Predicted years until full recovery ^a^
	2016/17	2017/18	2018/19	2016/19		
Lagoon	2.1	2.6	4.2	9.0*	3.0	0.7
West (shallow)	1.6	2.0	3.2	6.9*	2.3	4.8
East (shallow)	0.9	1.1	1.8	3.9*	1.3	8.5

Asterisks indicate significant increase between years (see Fig. 3a). Note that these were only calculated for locations where hard coral cover significantly increased based on the GEE analysis.

^a^Based on annual rate of change and assumes linear increase.

At the deep locations hard coral cover did not change between 2016 and 2019 (Fig. 3f). Although turf algae cover decreased between 2016 and 2018, it still covered 51% of the western benthos in 2019 (Fig. 3i), 70% more than pre-bleaching. At the east, turf algae cover remained below pre-bleaching levels but, similar to the shallow east, *Halimeda* and turf algae combined dominated the benthos at 53–61% and 9–23% cover between 2017 and 2019, respectively (Fig. 3i,j).

*Montipora* was the only hard coral genus that increased significantly between 2016 and 2019 (Supplementary Table S4, Supplementary Fig. S3). Although the absolute cover of *Montipora* was relatively low in 2019 at all shallow locations (< 3.5%), its increase contributed *ca.* 39% to the overall hard coral cover increase at the shallow west and east during 2016–2019 (Supplementary Table S5). The increase of ‘other hard corals’, albeit statistically not significant, contributed 38% (shallow east) and 29% (shallow west) to overall hard coral cover increase. In the lagoon, branching *Porites* and ‘other hard corals’ contributed most to overall hard coral cover increase between 2016 and 2019 (36% and 33%, respectively) although increases were not statistically significant. Although overall soft coral cover did not increase at any location, *Rhytisma* covered 7.0% of the benthos at the deep west in 2019 (vs. not being recorded in 2016), while remaining < 1.5% at all other locations (Supplementary Fig. S3).

**Figure 4:**
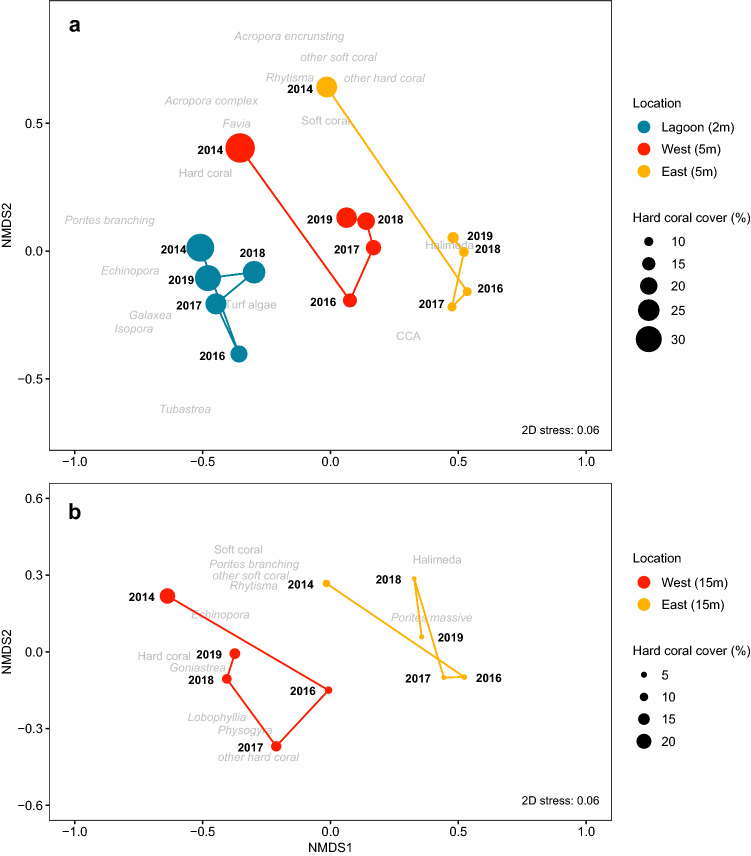
Non-metric multidimensional scaling (nMDS) of coral communities between 2014 and 2019 at Aldabra’s shallow (**a**) and deep (**b**) locations. Vectors connecting years display directional change of coral community composition at each location. Benthic and coral (italics) groups driving differences among locations and years displayed in grey. Scaled points indicate mean percent hard coral cover per location per year. Note that ‘other hard corals’ and ‘other soft corals’ contain different taxa than in the GEE analysis.

In terms of coral community composition (Fig. 4), in the lagoon, where bleaching-induced coral mortality was lowest and the magnitude of hard coral recovery was highest, coral community composition transitioned back to pre-bleaching assemblages between 2016 and 2019. Less pronounced than in the lagoon, western communities also showed returning transitions towards pre-bleaching assemblages, even at the deep reefs.

## Discussion

### Spatial variation in coral mortality following bleaching

Spatial variation in the extent of bleaching-induced hard coral mortality was clearly evident at Aldabra (see also Cerutti et al.^33^). Hard coral loss on the seaward reefs ranged between 51 and 62% and was only marginally lower at 15 m compared to 5 m water depth, indicating that corals in deeper water were as susceptible to heat stress as those in the shallows. This contrasts with other studies in which shallow coral communities suffered substantially higher post-bleaching mortality than deeper ones (e.g. Marshall and Baird^13^), but appears similar to observations in the Chagos Archipelago following the 1998 bleaching event^34^. In Aldabra’s lagoon, hard coral loss (34%) was considerably lower than on seaward reefs (51–62%). A lower reduction of hard coral cover inside Aldabra’s lagoon was also observed following the 1998/1999 bleaching event^35^, although no data is available for comparison. Within our study’s time frame, mean daily temperature variability was three-fold higher in the lagoon than on the seaward reefs, which has been proposed to result in inherently higher heat stress tolerance of hard corals and thus lower bleaching mortality^11,12,36^. Lagoon corals may also have been protected from UV radiation as a result of UV-absorbing leachate from seagrass leaves being steeped inside the lagoon^37^ or due to light attenuation from suspended particles^38,39^. Seagrass beds can be found in the vicinity of the lagoonal study reefs^40^ and (in comparison to the seaward reefs) turbidity in the lagoon was noted to be relatively high during surveys at slack high tide (pers. obs. Dec 2016, 2017, 2018, A.K.). Both mechanisms could result in reduced irradiance intensity experienced by corals, acting as additional buffers against thermal stress (but see Fisher et al.^41^). Furthermore, the high amount of suspended particulate material may allow corals to derive nutrients by heterotrophic feeding, contributing to higher bleaching survival when symbiont-acquired nutrients are low or entirely lacking^9,42^.

Across all locations, *Acropora* and *Montipora* consistently exhibited the highest susceptibility and suffered extensive losses following the bleaching event. Both genera are amongst the most susceptible hard corals to bleaching^13,43,44^ while massive *Porites*, as found elsewhere^45,46^, appeared resistant. However, the atoll-wide resistance of *I. palifera* and the location-specific resistance of branching *Porites* are striking (see also Cerutti et al.^33^). Whilst *I. palifera* remained abundant at the shallow western and lagoonal reefs, branching *Porites* (at Aldabra e.g. *P. cylindrica*, *P. harrisoni*, *P. monticulosa*, *P. profundus* and *P. rus*, which can exhibit branching or digitate growth forms^31^) suffered substantially lower mortality in the lagoon and remained relatively abundant there post-bleaching. Although it is currently unknown whether different species of branching *Porites* were present at Aldabra’s seaward reefs and in the lagoon, it is reasonable to suggest that adaptation to the variable lagoon environment was conducive to lower bleaching susceptibility and mortality.

### Post-bleaching trajectories

Aldabra’s reef trajectories in the three post-bleaching years varied substantially across locations. In 2019, hard coral cover at the shallow west (23%) and the lagoon (30%) reached 68% and 93% of the pre-bleaching cover, respectively (vs. 15% and 21% hard coral cover in 2016, i.e. 44% and 65% of pre-bleaching cover, respectively), and by 2018, benthic communities were no longer dominated by turf algae. Although coral recovery also occurred at the shallow east (from 9% hard coral cover, i.e. 38% of pre-bleaching cover in 2016 to 13% hard coral cover, i.e. 54% of pre-bleaching cover in 2019), *Halimeda* remained the single most dominant benthic group. There was no coral recovery at the deep locations, with reefs remaining dominated by turf algae (west) and *Halimeda* (east) between 2016 and 2019.

Coral recovery was particularly fast inside Aldabra’s lagoon, almost reaching pre-bleaching levels within 3 years. It is possible that a large proportion of hard corals in the lagoon experienced only partial mortality and were able to rapidly regrow post-bleaching. This seems especially likely because the coral community composition also nearly reassembled to pre-bleaching levels by 2019. Survival and growth of remnant coral colonies is an important component in reef recovery, specifically in the first few years post-disturbance when the reproductive capacity of corals may be low^23,47,48^. This process is particularly important for isolated reefs that rely on coral recruitment from local sources as was reported from the remote Scott Reef, Australia^23^ and the Inner Seychelles^3^. In both cases, recovery following the 1998/1999 bleaching event was slow for 7–10 years but then increased exponentially with increasing recruitment capacity. Given that recovery of Aldabra’s seaward reefs following the 1998/1999 bleaching event was slow until 2005^49^ (after which no further records are available), but coral cover had reached high levels by 2014, coral recruitment from local sources is likely important. The coral community in Aldabra’s lagoon may be critical for the long-term recovery of Aldabra’s reef system, with the potential to boost recovery at the seaward reefs. Nevertheless, information on the connectivity within Aldabra’s reefs as well as to other reefs in the region is limited (but see Crochelet et al.^50^) and research is needed in this area.

At both the lagoon and shallow west, the trajectories of turf algae and CCA following the bleaching event indicate a critical component of reef recovery^44^. Although the post-bleaching increase in CCA cover may be an artefact of the two-dimensional survey method employed here (i.e. where the loss of canopy forming corals may simply have increased the visibility of CCA underneath^51^), its stable cover in all post-bleaching years may benefit recovery processes. CCA can promote reef recovery by stabilising the reef framework^52^ and enhancing coral larvae settlement^26,27^, whereas turf algae, often the first benthic group to grow over dead coral substrate following disturbance^20,23,53,54^, can aggressively compete with adult corals through smothering and allelopathy^52^, and can inhibit coral larval settlement^26,55^ and the survival of coral recruits^56^. The rapid proliferation of turf algae following bleaching events can thus be detrimental to hard coral recovery. At Aldabra’s shallow reefs, however, turf algae cover was reduced to pre-bleaching levels within 2 years after bleaching (by 2018). This rapid reduction and the general lack of fleshy macroalgae across Aldabra’s reefs indicate high grazing pressure. Indeed, Aldabra hosts the highest biomass of herbivorous fish in the Seychelles^31^ and herbivory may actually have facilitated an increase in CCA^52^ following the bleaching event. Both herbivory and CCA abundance may be positively influenced by nutrient input of the guano from Aldabra’s numerous seabird populations^57^, as was recently shown at the Chagos Archipelago^58^.

At the shallow east and the deep reefs, recovery is likely restricted by abiotic conditions. Robinson et al.^4^ found that coral recovery in the Inner Seychelles following the 1997/1998 bleaching event was prolonged with increasing water depth and wave exposure which was attributed to lower coral growth rates, e.g. due to increased light attenuation at depth^59,60^. In terms of wave exposure, coral community composition is naturally shaped along wave energy gradients^24,61,62^. Branching (i.e. usually fast-growing) hard corals are less likely to occur where wave energy is high, which ultimately prolongs the time for hard coral recovery in those areas^4^. These abiotic conditions likely reflect the lack of hard coral recovery at Aldabra’s deep reefs (depth) and the lower magnitude of recovery at the shallow east (wave exposure). Although Aldabra’s deep western reefs remain dominated by turf algae, hard coral recovery can still occur in the long term. However, as already noted by Drew^63^ and Stobart et al.^35^, there is a prominent gradient of decreasing coral cover and increasing *Halimeda* cover from the western towards the eastern seaward reefs, attributed to an increase in hydrodynamic energy. *Halimeda* was already present in high abundance on Aldabra’s eastern reefs pre-bleaching and remained unchanged until 2016. However, by 2017 it became the single most dominant benthic category in the east, reaching up to 61% of overall benthic cover by 2018, potentially further constraining coral recovery there.

Hard coral recovery following disturbance is often driven by *Acropora* and *Pocillopora*^4,64–68^, but also other taxa such as *Porites*, *Montipora*, *Isopora*, *Galaxea* and *Pavona* are named in the literature^64,67,69–71^. At Aldabra’s lagoon, hard coral cover increase was largely due to the contributions of branching *Porites* and ‘other hard corals’. Many taxa that exhibit branching growth forms are characterised by life history traits that favour fast growth and wide dispersal, and are therefore often the first to recover at recently disturbed habitats. However, at the seaward reefs, most taxa that exhibit branching growth forms remained scarce during 2016–2019, with encrusting *Montipora* and ‘other hard corals’ contributing most to the early overall hard coral recovery. Previously, fast recovery of encrusting *Montipora* was associated with higher wave exposure^20^, which may explain why it is mainly found at Aldabra’s seaward locations.

Due to different bleaching susceptibility and recovery potential of coral taxa, overall hard coral recovery can be accompanied by a shift in coral community composition^6,72^ (but examples for reassembly also exist^6,23,72^). Such community shifts can alter the ecological functions of a reef and their response to future disturbance^6^. At Aldabra, coral communities in the lagoon recovered and reassembled almost completely within 3 years and also the western communities showed reassembly trajectories until 2019. The lower post-bleaching mortality of branching *Porites* in the lagoon and its relative contribution to overall hard coral recovery possibly indicates location-specific resilience, which may also have been important for the rapid return to pre-bleaching coral community composition there. As reef recovery is ongoing at Aldabra’s seaward reefs, however, it is possible that further taxa emerge which could dominate hard coral recovery, particularly if coral recruitment speeds up coral recovery^3,23^.

Furthermore, in contrast to the lagoon, pre-bleaching coral communities at the seaward reefs were not only characterized by hard corals. Soft corals also constituted an important component at the seaward reefs prior to the bleaching event and community reassembly there also depends on soft coral recovery. Overall soft coral recovery was negligible during 2016–2019, however, *Rhytisma* covered 7% of the benthos at the deep west in 2019, albeit not being recorded in 2016. This is similar to observations at Aldabra following the 1998 bleaching event where *Rhytisma* increased at 10 m water depth from zero to 8% cover by 2002, i.e. during the same time frame as studied here^49,73^. The subsequent increase to 26% cover by 2004 and the slow hard coral recovery caused reason to suggest Aldabra’s reefs had undergone a shift from hard to soft coral dominance^32^. It is possible that *Rhytisma* rapidly re-gains its previous abundance, with potential negative implications for hard coral recovery, but unlike implied previously^32,74^, the high abundance of *Rhytisma* has been restricted to Aldabra’s deep western reefs and does not affect the entire reef system.

### Aldabra’s reef recovery in context

Overall annual rate of change in absolute hard coral cover at Aldabra’s shallow reefs was 2.2% over 3 years (Supplementary Table S6), similar to values reported for reefs within and outside no-take marine protected areas in Kenya (2.3% over 3 years), the Maldives (2.3% over 4 years) and Palau (2.1% over 8 years). Higher annual increases during similar recovery time frames were reported from Alphonse Atoll, Seychelles (2.9% over 2 years) and the Lakshadweep Islands (3.5% over 3 years), both of which are unprotected. In the Inner Seychelles, overall hard coral cover increased annually by 1.8% over 9 years (including reefs within and outside no-take marine protected areas) and at the remote Chagos Archipelago, cover increased annually by 2.6% over 11 years. However, looking at individual reefs regardless of location and level of protection, 56% of the annual rates of change provided in Supplementary Table S6 fall within the range reported for Aldabra’s reefs (1.3–3.0%), with 21% and 23% of the values falling below or above that range, respectively (Supplementary Figure S4). Hard coral cover increase at Aldabra’s shallow reefs, in terms of annual rate of change, was therefore not exceptionally low or high in comparison to reefs elsewhere.

Graham et al.^5^ reviewed global recovery rates following various acute disturbances and found a mean annual rate of 3.56% coral cover increase, with a range from 0.13 to 12.49%. However, rates were lowest in fully protected areas (0.8% vs. 4.0% in open areas) and varied depending on post-disturbance coral cover (between 2 and 4%). It was proposed that because protected areas promote higher coral cover (provided they are undisturbed), they are more susceptible to disturbances such as coral bleaching and may lose a higher proportion of corals than unprotected areas (the authors noted however, that enforcement or compliance could not be standardised among the assessed protected areas). Arguing that recovery dynamics are likely nonlinear and may be faster where disturbances have opened up more space for coral recruitment, recovery from high to medium coral loss (in their study 6–20% post-disturbance cover) may be faster than from extreme (< 5% post-disturbance cover) and small losses (21–30% post-disturbance cover)^5^. Our results do not match these findings (i.e. at Aldabra, recovery rate was highest where coral loss was lowest), probably because the early recovery at Aldabra observed here, particularly in the lagoon, is likely driven by the growth of remnant coral colonies rather than coral recruitment. Furthermore, in contrast to the studies used by Graham et al.^5^, full recovery is not realised at all of Aldabra’s locations due to the short recovery time frame studied here. These discrepancies show that post-disturbance recovery measured by hard coral cover increase alone misses the complexities of this process^5^ and reduces the meaningfulness of comparisons if no context is provided.

The return of coral cover and coral community composition to pre-disturbance values can provide this context and are useful indicators. For example, Aldabra’s lagoon nearly completely recovered its pre-bleaching hard coral cover within 3 years, matched by only two other reefs in our comparison within a similar time frame for recovery (Vipingo and Kanamai in Kenya, both unprotected; Supplementary Table S6). However, despite these often being the best indicators at data scarce locations (if available at all) it is important to acknowledge that longer-term effects of reef degradation may be masked and that reef state immediately pre-disturbance is likely not the ideal baseline (i.e. shifting baselines^75^).

### Future prospects for Aldabra’s reefs

Despite its remoteness and strict protection, Aldabra’s reefs were significantly impacted by the 2015/2016 bleaching event, joining other remote reef systems such as the Chagos Archipelago^54^ and the northern Great Barrier Reef^76^ that suffered extensive bleaching-induced coral mortality. Nevertheless, we show that Aldabra’s lagoon rapidly recovered its pre-bleaching coral cover and reassembled to its pre-bleaching coral community composition within 3 years. While at the shallow seaward reefs, coral recovery is predicted to take *ca*. 5–9 more years (if there is no major bleaching event), it is likely prolonged at the deep reefs.

Our results add to the work of Cerutti et al.^33^ in providing locally important baseline information for ongoing coral reef research, ultimately feeding into Aldabra’s management and corroborating its protective status. On the global level, our study adds to previous work conducted at remote reefs (e.g. Gilmour et al.^23^, Sheppard et al.^34^), advancing the knowledge of coral bleaching impact and recovery in the absence of direct human disturbance. With an expected increase in the magnitude and frequency of mass bleaching events^2^, intervals between major events will become too short for adequate reef recovery, and reef locations where bleaching impact has been low so far, are likely to become more vulnerable to temperature stress. Our research underlines the need for drastic measures to reduce greenhouse gas emissions, alongside continued reduction and management of local human disturbance to conserve the world’s coral reefs.

## Methods

### Study site

**Figure 5:**
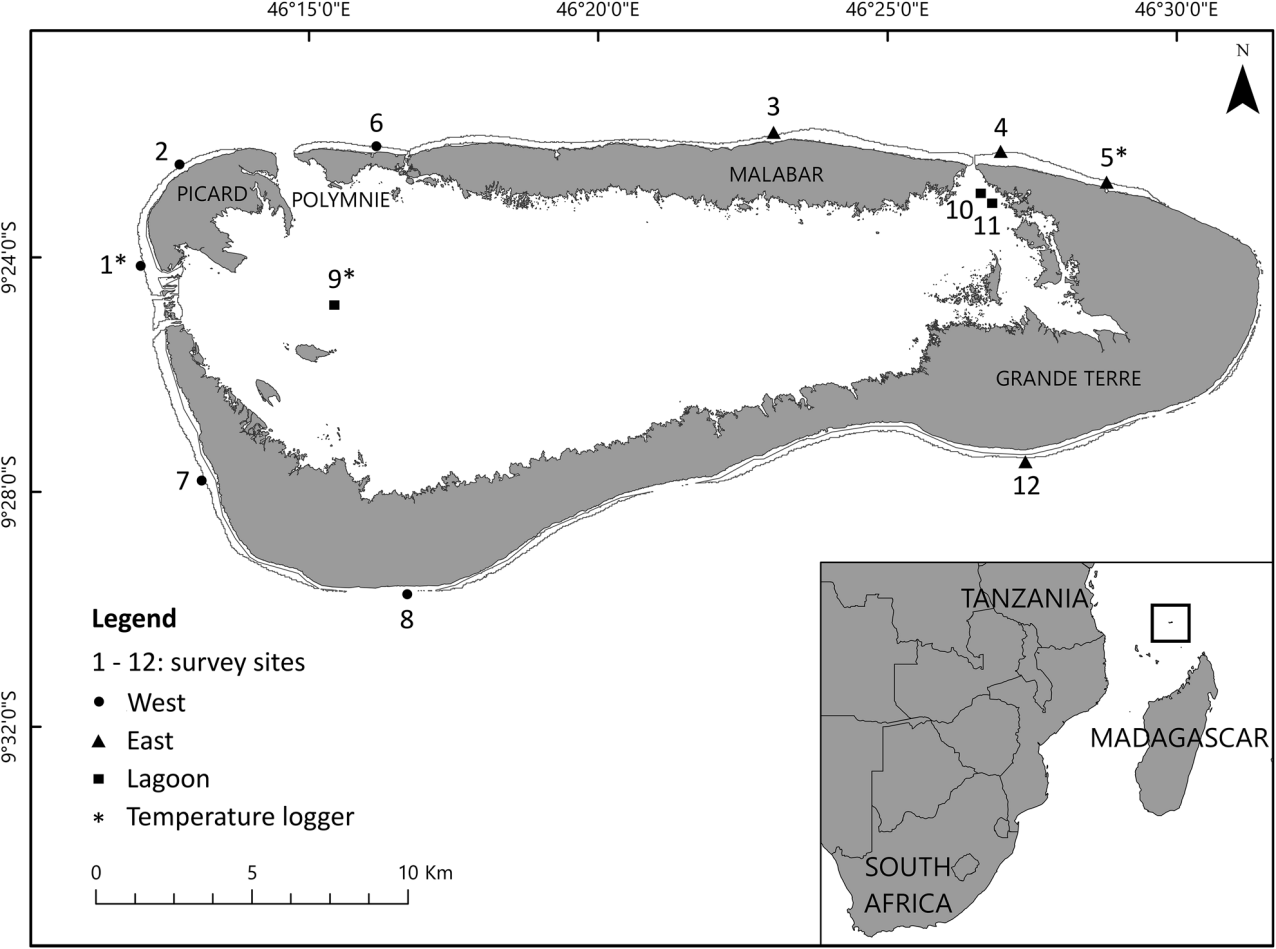
Location of Aldabra Atoll in the Western Indian Ocean (inset) with its four main islands, 12 survey sites at the seaward west (five sites), east (four sites) and in the lagoon (three sites) and three temperature logger sites (modified from Cerutti et al.^33^).

Aldabra (46° 20′ E, 9° 24′ S), managed by a Public Trust, the Seychelles Islands Foundation (SIF), since 1979, is an elevated coral atoll in the southwest of the Seychelles archipelago spanning 34 × 14.5 km (Fig. 5). Two distinct seasons govern Aldabra’s climate; the south-east trade winds from April to November create a dry and cooler climate whilst the north-west monsoon from November/December to March generates wet and warmer conditions. Aldabra’s large lagoon (196 km^2^) is encircled by four main islands and subject to a 2–3 m tidal range^77^. The north-east, east and south-east parts of Aldabra are exposed to strong winds and high wave energy, whilst the north-west and west are relatively sheltered^78^. Because of this contrasting level of exposure, we divided Aldabra’s reefs into three locations: seaward western, seaward eastern and lagoonal reefs (contrasting with Cerutti et al.^33^ who did not make the distinction between west and east at the seaward reef).

During the 2014–2017 Global Scale Coral Bleaching Event^79^, Aldabra’s reefs experienced continuous bleaching risk between December 2015 and June 2016. Satellite derived sea surface temperature reached a maximum of 30.7 °C in March 2016, resulting in a peak degree heating weeks (DHW^80^) value of 3.4 °C-weeks^33^ (but note that the NOAA Aldabra virtual station is located *ca.* 55 km north-east of Aldabra: 46° 50′ E, 9° 00′ S).

### Data collection

Benthic data was collected from 12 permanent study sites (Fig. 5) once prior to bleaching, and four times post-bleaching. We refer to the survey periods as follows: (1) 2014 (pre-bleaching; data collected Dec 2014–Jan 2015), (2) 2016 (1st year post-bleaching; Dec 2016), (3) 2017 (2nd year post-bleaching; Dec 2017), (4) 2018 (3rd year post-bleaching; Dec 2018–Jan 2019) and (5) 2019 (4th year post-bleaching, Nov 2019–Jan 2020).

Of the 12 study sites, permanently marked transects are located at 5 and 15 m water depth at nine seaward survey sites (n = 18 seaward transects) and at *ca*. 2 m water depth at three sites in the lagoon (n = 3 lagoonal transects) (Fig. 5). All permanent transects are 50 m long, follow the depth contour, and are parallel to the shoreline. In 2014, two transect sections at 0–10 m and 20–30 m were surveyed on each permanent transect. During all post-bleaching surveys, increased availability of resources allowed an additional section to be surveyed on each permanent transect at 40–50 m. Along each section, benthic photoquadrats were collected on both sides of the tape measure with a GoPro camera attached to a 70 × 50 cm PVC frame at 70 cm height (pre-bleaching: GoPro Hero-3 Silver, 11 megapixels; post-bleaching: GoPro Hero-4 Silver and GoPro Hero-5 Black, 12 megapixels).

Water temperature data was obtained from three Onset loggers (HOBO U22-001) deployed between 2015 and 2018 at three of the permanent sites representing conditions in the lagoon (Site 9, 2 m water depth), shallow west (Site 1, 5 m) and shallow east (Site 5, 5 m; Fig. 5). Water temperature was recorded every 30 min with an accuracy of ± 0.2°C^81^.

### Data processing and statistical analysis

R version 3.6.1^82^ was used for statistical analysis. All graphs were created with the *ggplot2* package^83^.

Benthic photos were analysed using Coral Point Count with Excel extensions (CPCe)^84^ by identifying the benthos at 16 randomly assigned points per image as described by Cerutti et al.^33^. This yielded a total of 179,247 points (excluding points on photoquadrat frame, transect tape or shadow) across all survey years. Five major categories were assigned for benthic cover analysis: hard coral, soft coral, turf algae, CCA and *Halimeda* spp. (hereafter ‘*Halimeda*’), which was by far the most dominant macroalga on Aldabra’s reefs. Fleshy macroalgae was not included as a category in the analysis as its mean cover was low across all locations and years (0–1.5%), except in 2018 at the deep eastern reefs (9.8%) where a seasonal *Caulerpa racemosa* bloom was recorded in Dec 2018 which had subsided by Feb 2019 (Supplementary Table S7), For the coral community, nine taxonomic categories were chosen following Cerutti et al.^33^ whereby the category ‘Acroporidae (excluding *Isopora palifera*)*’* was replaced by *Acropora* and *Montipora* for more detailed analysis: *Acropora* (branching, plating and encrusting growth forms combined), *Montipora* (encrusting), *I. palifera*, Merulinidae, branching *Porites* (also includes digitate growth forms), massive *Porites,* other hard corals, *Rhytisma,* and other soft corals. To assess changes of the selected categories across locations between 2014 and 2016 (bleaching impact), the mean percent benthic cover of two transect sections was used as a response variable due to no third transect sections being conducted in 2014. To evaluate changes at these locations between 2016 and 2019 (post-bleaching trajectories), the mean percent benthic cover of three transect sections was included as a response variable.

Generalised Estimating Equations (GEE—‘geeglm’ function, *geepack* package^85^) with auto-regressive correlation structure were used to test for differences in the benthic and coral cover categories across time (bleaching impact: 2014, 2016; post-bleaching trajectory: 2016, 2017, 2018, 2019) and location (lagoon, west, east) at shallow (2 m, 5 m) and deep (15 m) water depth. Fixed explanatory variables included year, location, and their interactive effect (i.e. model structures: Year × Location; Location + Year; Location; Year). Survey site was set as a random factor to correct for pseudo-replication (transects sections). To correct for non-normality of the response variable (percentage benthic cover), we used different error distributions and link functions that best fitted the models and depended on the nature of the data (see Supplementary Tables S1–S4 for further details). We validated the models by running Generalised Linear Models first (‘glm’ function, R *stats* package^82^) and checking the residual distribution to see if the assumption of homogeneity of variance, normality and leverage were met. We then also checked the Pearson residual distribution for the GEE models. We used a post-hoc analysis based on least square means with Bonferroni adjustment (‘lsmean’ function, *lsmean* package^86^) to identify pairwise differences between the variables in significant interactive models (Year × Location). Due to many low values of *Rhytisma* (shallow and deep) and all coral categories at deep locations between 2016 and 2019, models of post-bleaching trajectories had a poor fit and these categories could not be tested statistically. All summaries of the GEE model outputs are provided in Supplementary Table S8 (separate file).

To further assess reef recovery, we calculated: (1) the annual rate of change in absolute hard coral cover increase^87^ and (2) an estimate of years remaining (from 2019) until hard coral cover reaches pre-bleaching levels (see Eqs. 1 and 2 in Supplementary Material). Acknowledging the latter as an extremely simplified projection that assumes a linear increase in hard coral cover, we applied both calculations only to those locations where hard coral cover had increased significantly between 2016 and 2019 (i.e. according to GEE analysis).

To assess which hard coral categories contributed most to overall hard coral cover increase (where it was significant according to GEE analysis), we calculated the absolute change in percentage cover between 2016 and 2019 for each hard coral category. Based on this, we calculated the contribution of each coral category to overall hard coral cover increase.

To visualise coral community trajectories, non-metric multidimensional scaling (nMDS) based on Bray–Curtis dissimilarity matrices of the coral community (using percent cover) was performed (‘metaMDS’ function, *vegan* package^88^). Only coral categories covering ≥ 5% of any transect section were included in the analysis. All other categories were combined into ‘other hard corals’ and ‘other soft corals’ (note that these categories contain different taxa to ‘other hard corals’ and ‘other soft corals’ in the GEE analysis). To display which coral and major benthic categories correlated with the community differences, significant correlation vectors were overlaid on nMDS plots (‘envfit’ function).

To assess daily water temperature regimes, overlapping time series of temperature data logged at the three monitored sites (Sites 1, 5 and 9) were selected (Feb 2015 and Nov 2018). Due to technical issues there were data gaps of 1-week (2–9 Apr 2015) and 5-months (12 Dec 2016–5 May 2017) for Site 1 and a data gap of 3-weeks for Site 9 (10 Dec 2016–1 Jan 2017). At Site 5, data was only available from Feb 2015 to Apr 2017 due to logger loss. Across the entire period, a minimum of 809 days remained for each logger (Site 9: 1353; Site 1: 1248, Site 5: 809) from which annual mean daily temperature (mean, maximum, minimum) and temperature variability (range, coefficient of variation) was calculated. To test for differences in these measures between sites, two-sided t-tests were conducted for each combination (i.e. Site 1 vs. Site 9; Site 1 vs. Site 5; Site 5 vs. Site 9) with Bonferroni adjustment applied to *p* values to correct for multiple comparisons.

### Aldabra’s coral recovery in global context

To gain perspective on how Aldabra’s reef recovery fits into the global context, a table of studies reporting reef recovery was compiled (Supplementary Table S6) and the annual rate of change in absolute hard coral cover (Eq. 1 in Supplementary Material)^87^ was calculated for each reef. Studies were drawn from Baker et al.^89^ and Graham et al.^5^ and supplemented by more recent literature. Only studies reporting uninterrupted recovery from bleaching events were included; i.e. where no additional acute disturbance (e.g. bleaching, storm, *Acanthaster* outbreak) was reported within the recovery period.

## Supplementary information

Supplementary file1

Supplementary file2

## Data Availability

The datasets generated and analysed during the current study are available from the corresponding author on reasonable request.
