# Supplementary material for: Early trajectories of benthic coral reef communities following the 2015/16 coral bleaching event at remote Aldabra Atoll, Seychelles
Source: Sci Rep. 2020 Oct 12;10:17034. doi: 10.1038/s41598-020-74077-x (PMC7550576; doi:10.1038/s41598-020-74077-x)
Supplement: Supplementary file 1 — Supplementary file1 [file 41598_2020_74077_MOESM1_ESM.pdf]

Supplementary information:

Early trajectories of benthic coral reef communities following the 2015/16 coral bleaching event at remote Aldabra Atoll, Seychelles

Anna Koester[1], Valentina Migani[2], Nancy Bunbury[3,4], Amanda Ford[5], Cheryl Sanchez[3], Christian Wild[1]

[1]Marine Ecology Department, Faculty of Biology & Chemistry, University of Bremen, Leobener Straße 6, 28359 Bremen, Germany

[2]Institute for Ecology, Faculty of Biology & Chemistry, University of Bremen, Leobener Straße 5, 28359 Bremen, Germany

[3]Seychelles Islands Foundation, PO Box 853, Victoria, Mahé, Seychelles

[4]Centre for Ecology and Conservation, University of Exeter, Cornwall Campus, Penryn, TR10 9FE, UK

[5]School of Marine Studies, Faculty of Science, Technology & Environment, University of the South Pacific, Suva, Fiji

This file includes (in order of first appearance in paper):

- Table S1–S2: Bleaching impact on benthic and coral categories - Output of Generalised Estimating Equations model comparisons
- Figure S1: Cover of coral categories in 2014 and 2016
- Figure S2: Annual mean daily water temperature (mean, maximum, minimum) and coefficient of variation
- Table S3–S4: Post-bleaching trajectories of benthic and coral categories - Output of Generalised Estimating Equations model comparisons
- Figure S3: Cover of coral categories between 2016 and 2019
- Table S5: Absolute percentage change in mean cover of coral taxa between 2016–2019 at shallow locations
- Table S6: Post-bleaching hard coral recovery of 52 reefs at 11 locations worldwide
- References to Table S6
- Figure S4: Histogram of annual rates of change of reefs displayed in Table S6
- Table S7: Cover of fleshy macroalgae
- Equation 1 and 2: Annual rate of change in absolute hard coral cover (equation 1) and estimate of years remaining until hard coral cover reaches pre-bleaching levels (equation 2)

Table S1: Bleaching impact on benthic categories. Effect of location (western seaward, eastern seaward, lagoon) and year (2014, 2016) on selected benthic categories at shallow and deep reefs. Degrees of freedom (dF), chi-square-value ($\chi2$) and *p*-value obtained from GEE model comparisons with ANOVA (type I). Where two error distributions are shown, grey coloured text corresponds to the analysis of deep locations. Significance levels: * $p < 0.5$; ** $p < 0.01$; *** $p < 0.001$; ns = not significant: $p > 0.05$. CCA = crustose coralline algae.

			shallow			deep		
Category	Error distribution (link function)	Fixed factor	dF	$\chi2$	*p*	dF	$\chi2$	*p*
Hard coral	Gamma (inverse)<br>Gamma (log)	Location	2	3.6	ns	1	4.0	*
		Year	1	10.9	***	2	7.5	**
		Location:Year	2	6.21	*	2	1.2	ns
Soft coral	Gamma (log)	Location	2	10.1	**	1	1.8	ns
		Year	1	31.6	***	2	27.9	***
		Location:Year	2	2.3	ns	2	1.5	ns
CCA	Gamma (log)	Location	2	5.6	ns	1	0.6	ns
		Year	1	11.9	**	2	33.9	***
		Location:Year	2	1.6	ns	2	0.3	ns
Turf algae	Gamma (log)	Location	2	40.6	***	1	>0.0	ns
		Year	1	5.6	*	2	3.2	ns
		Location:Year	2	2.3	ns	2	4.5	*
Halimeda	Binomial (logit)	Location	2	14.9	***	1	11.4	***
		Year	2	0.1	ns	2	>0.0	ns
		Location:Year	1	13.2	**	2	1.9	ns

Table S2: Bleaching impact on coral categories. Effect of location (western seaward, eastern seaward, lagoon) and year (2014, 2016) on selected coral categories at shallow and deep reefs. Degrees of freedom (dF), chi-square-value ($\chi 2$) and p-value obtained from GEE model comparisons with ANOVA (type I). Where two error distributions are shown, grey coloured text corresponds to the analysis of deep locations. Significance levels: * $p < 0.5$; ** $p < 0.01$; *** $p < 0.001$; ns = not significant: $p > 0.05$.

Category	Error distribution (link function)	Fixed factor	shallow			deep		
			dF	$\chi 2$	*p*	dF	$\chi 2$	*p*
Acropora	Gamma (log)	Location	2	13.5	***	1	3.9	*
		Year	1	31.6	***	1	17.1	***
		Location:Year	2	1	ns	1	1.6	ns
Montipora	Gamma (log)	Location	2	14.5	***	1	0.7	ns
		Year	1	14.2	***	1	31.5	***
		Location:Year	2	2.9	ns	1	0.1	ns
Isopora palifera	Gamma (log)	Location	2	10.2	**	1	0.5	ns
		Year	1	1	ns	1	0.1	ns
		Location:Year	2	12.9	**	1	0.3	ns
Merulinidae	Gamma (log)	Location	2	7.9	*	1	9.2	**
		Year	1	8.9	**	1	10.6	**
		Location:Year	2	0.2	ns	1	>0.0	ns
Porites massive	Gamma (log)<br>Gamma (inverse)	Location	2	2.3	ns	1	3.2	ns
		Year	1	0.7	ns	1	0.0	ns
		Location:Year	2	1.2	ns	1	6.7	*
Porites branching	Gamma (log)	Location	2	61.2	***	1	1.6	ns
		Year	1	143	***	1	16.2	***
		Location:Year	2	99.9	***	1	>0.0	ns
other hard corals	Binomial (logit)	Location	2	1.2	ns	1	2.4	ns
		Year	1	3.7	ns	1	1.8	ns
		Location:Year	2	2.8	ns	1	0.3	ns
Rhytisma	Gamma (inverse)<br>Gamma (log)	Location	2	122	***	1	9.0	**
		Year	1	6.1	*	1	925	***
		Location:Year	2	5.7	ns	1	8.0	**
other soft corals	Gamma (log)	Location	2	13.3	**	1	0.7	ns
		Year	1	19.6	***	1	6.9	**
		Location:Year	2	0.4	ns	1	0.6	ns

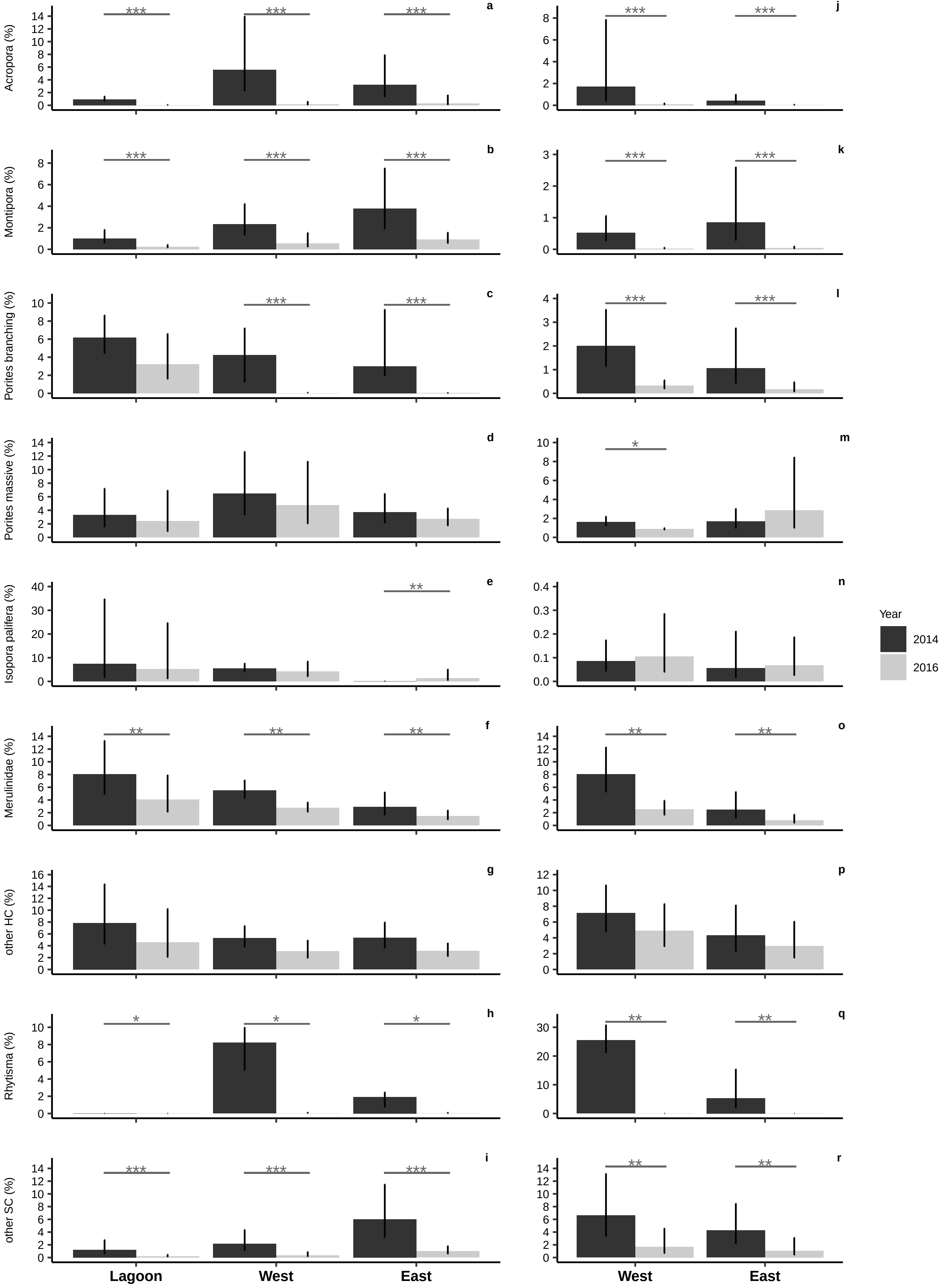

Figure S1: Mean cover of coral taxa at shallow (a–i) and deep locations (j–r) between 2014 and 2016 at Aldabra (transect sections: lagoon n = 6, west n = 10 per depth, east n = 8 per depth). Bars represent back−transformed estimates of GEE analysis with 95% confidence interval. Significant differences across years are indicated with asterisks (* p < 0.5; ** p < 0.01; *** p < 0.001)

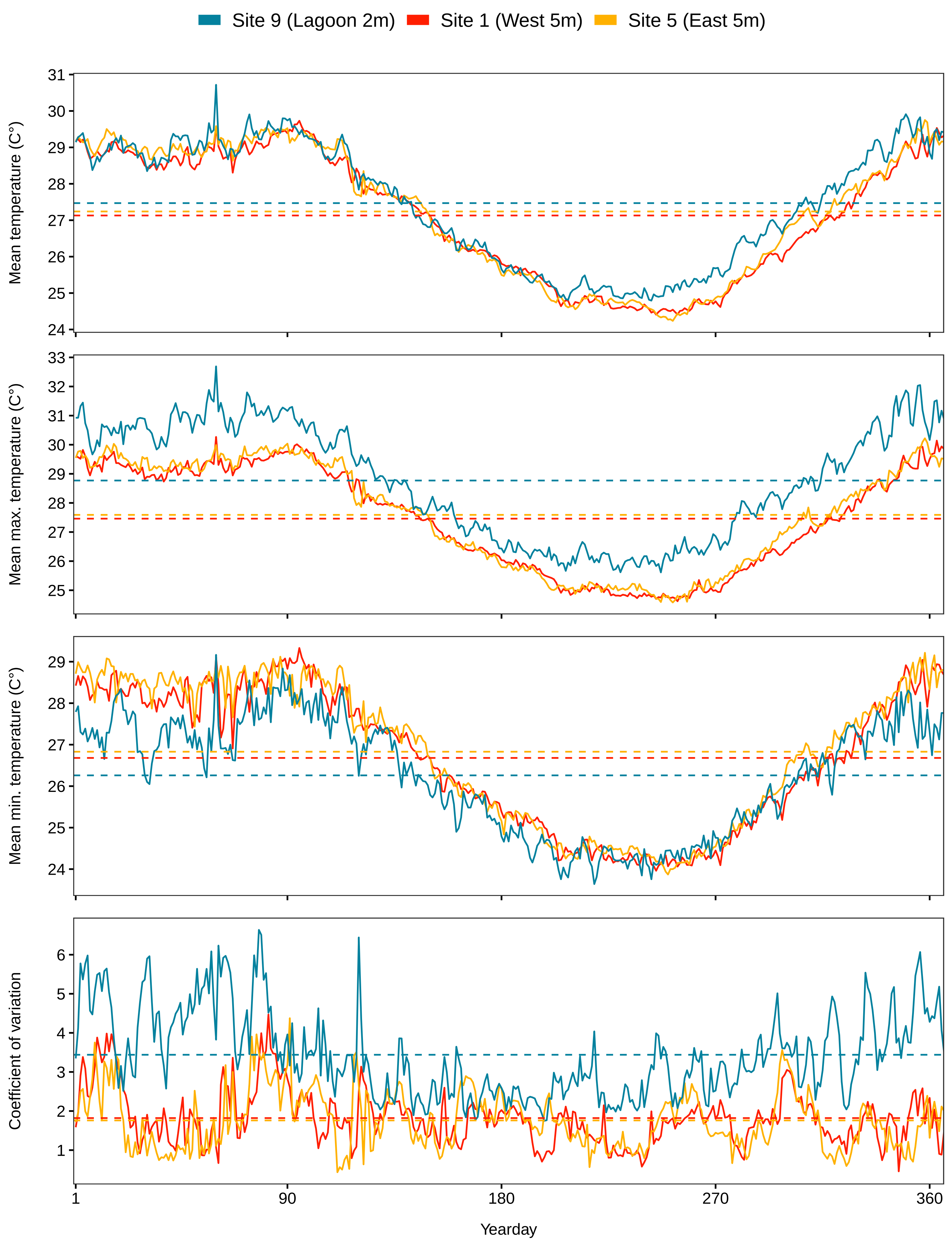

Figure S2: Annual mean daily water temperature (mean, maximum, minimum) and coefficient of variation at three representative sites in the lagoon and at the seaward west and east of Aldabra. Solid lines indicate daily means calculated from temperature records obtained in 30−min intervals between February 2015 and November 2018 (see methods). Dashed lines represent the mean within the study period.

Table S3: Post-bleaching trajectories of benthic categories. Effect of location (western seaward, eastern seaward, lagoon) and year (2016, 2017, 2018, 2019) on selected benthic categories at shallow and deep reefs. Degrees of freedom (dF), chi-square-value ($\chi 2$) and *p*-value obtained from GEE model comparisons with ANOVA (type I). Significance levels: * $p < 0.5$; ** $p < 0.01$; *** $p < 0.001$; ns = not significant: $p > 0.05$. CCA = crustose coralline algae.

			shallow			deep		
Category	Error distribution (link function)	Fixed factor	dF	$\chi 2$	*p*	dF	$\chi 2$	*p*
Hard coral	Gamma (log)	Location	2	97.2	***	1	28.4	***
		Year	3	15.2	**	3	0.5	ns
		Location:Year	6	1.1	ns	3	3.2	ns
Soft coral	Gamma (log)	Location	2	36	***	1	11.1	***
		Year	3	2.3	ns	3	3.0	ns
		Location:Year	6	5.2	ns	3	3.3	ns
CCA	Gamma (log)	Location	2	3.4	ns	1	16	**
		Year	3	3.4	ns	3	6.1	ns
		Location:Year	6	14.4	*	3	10.0	ns
Turf algae	Gamma (log)	Location	2	34.7	***	1	29.7	***
		Year	3	19.7	***	3	9.5	*
		Location:Year	6	4.6	ns	3	10.3	*
Halimeda	Binomial (logit)	Location	2	72.9	***	1	85.3	***
		Year	3	4.9	ns	3	4.6	ns
		Location:Year	6	3.0	ns	3	5.3	ns

Table S4: Post-bleaching trajectories of coral categories. Effect of location (western seaward, eastern seaward, lagoon) and year (2016, 2017, 2018, 2019) on selected coral categories at shallow reefs. Degrees of freedom (dF), chi-square-value ($\chi 2$) and p-value obtained from GEE model comparisons with ANOVA (type I). Significance levels: * $p < 0.5$; ** $p < 0.01$; *** $p < 0.001$; ns = not significant: $p > 0.05$. All categories from deep locations and *Rhytisma* from shallow locations had poor model fit and could not be tested statistically.

Category	Error distribution (link function)	Fixed factor	dF	$\chi 2$	*p*
Acropora	Binomial (logit)	Location	2	6.7	*
		Year	3	0.4	ns
		Location:Year	6	3.8	ns
Montipora	Binomial (logit)	Location	2	14.2	***
		Year	3	16.5	***
		Location:Year	6	2.1	ns
Isopora palifera	Binomial (logit)	Location	2	10.2	**
		Year	3	1.1	ns
		Location:Year	6	0.3	ns
Merulinidae	Binomial (logit)	Location	2	10.1	**
		Year	3	1.4	ns
		Location:Year	6	3.3	ns
Porites massive	Binomial (logit)	Location	2	9.6	**
		Year	3	>0.0	ns
		Location:Year	6	0.3	ns
Porites branching	Binomial (logit)	Location	2	69.6	***
		Year	3	5.6	ns
		Location:Year	6	0.1	ns
other hard corals	Binomial (logit)	Location	2	10.2	**
		Year	3	5.1	ns
		Location:Year	6	3.4	ns
other soft corals	Binomial (logit)	Location	2	4.3	ns
		Year	3	2.2	ns
		Location:Year	6	6.1	ns

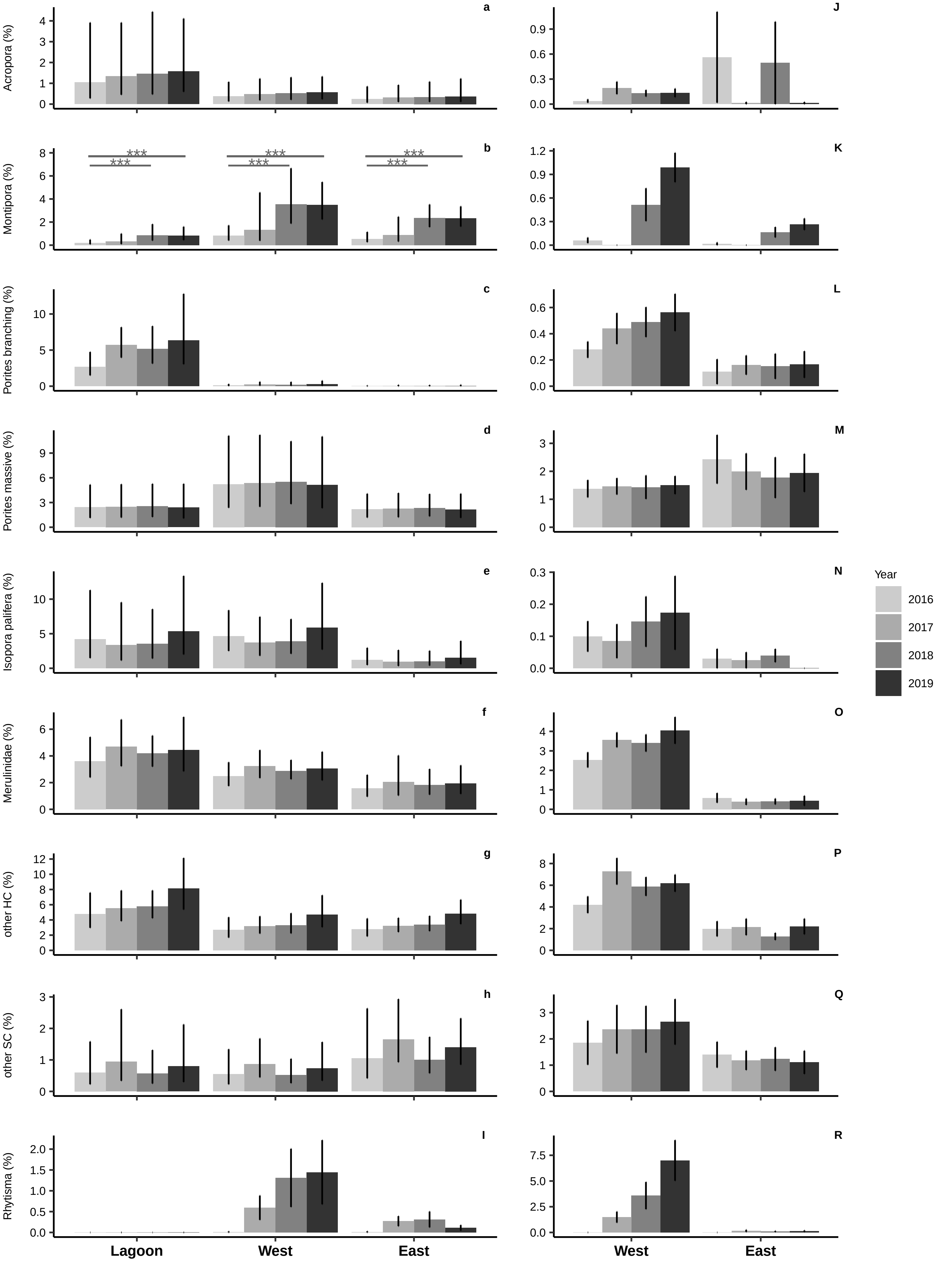

Figure S3: Mean cover of coral taxa at shallow (a–I) and deep locations (J–R) between 2016 and 2019 at Aldabra (transect sections: lagoon n = 9, west n = 15 per depth, east n = 12 per depth). Bars in plots a–h represent back−transformed estimates of GEE analysis with 95% confidence interval. Significant differences across years are indicated with asterisks (*** p < 0.001). Categories in plots I–R were not statistically tested due to poor model fit and are displayed as mean cover (± SE).

Table S5: Absolute percentage change in mean cover of coral taxa between 2016–2019 at Aldabra's shallow locations and the percent contribution to the change in overall hard coral cover. Taxa are sorted in descending order based on their contribution to overall change in hard coral cover. Note that values were obtained from back-transformed estimates of GEE analysis as displayed in Fig. S3, overall values displayed here (*) therefore slightly deviate from those obtained from GEE analysis of overall hard coral cover as displayed in Figure 4 and Table 2.

Location	Taxa	Mean cover (%)		Absolute Change (%)	Contribution to overall change (%)	Cumulated contribution (%)
		2016	2019			
Lagoon	*Porites* branching	2.68	6.35	3.67	36.06	36.06
	other hard coral	4.77	8.15	3.38	33.18	69.24
	Isopora palifera	4.18	5.34	1.15	11.32	80.56
	Merulinidae	3.61	4.46	0.85	8.34	88.91
	Montipora	0.20	0.84	0.64	6.32	95.23
	Acropora	1.05	1.58	0.53	5.16	100.39
	Porites massive	2.44	2.40	-0.04	-0.39	100.00
	Overall*	18.93	29.11	10.19	100.00	
West shallow	*Montipora*	0.83	3.50	2.67	39.38	39.38
	other hard coral	2.72	4.71	1.99	29.36	68.75
	Isopora palifera	4.63	5.90	1.27	18.72	87.47
	Merulinidae	2.48	3.07	0.59	8.71	96.18
	Acropora	0.37	0.56	0.19	2.77	98.95
	Porites branching	0.10	0.25	0.15	2.23	101.18
	Porites massive	5.23	5.15	-0.08	-1.18	100.00
	Overall*	16.37	23.14	6.78	100.00	
East shallow	other hard coral	2.78	4.81	2.03	43.76	43.76
	Montipora	0.55	2.32	1.78	38.31	82.07
	Merulinidae	1.57	1.95	0.38	8.19	90.26
	Isopora palifera	1.19	1.53	0.34	7.37	97.63
	Acropora	0.24	0.37	0.12	2.65	100.28
	Porites branching	0.01	0.03	0.02	0.36	100.65
	Porites massive	2.20	2.17	-0.03	-0.65	100.00
	Overall*	8.54	13.18	4.64	100.00	

Table S6: Post-bleaching hard coral recovery of 52 reefs at 11 locations worldwide. Overall values at each location are grand means obtained from reef level data (unless given in reference). Locations are sorted in ascending order by number of recovery years.

Location (bleaching event)	Pre-bleaching mean cover (%)	First record post-bleaching		Last record post-bleaching		No recovery years	Recovery (%)[b]	Annual rate of change (%)	Dominant recovery taxa	Level of protection (year of designation)
		Year	Mean cover (%)	Year	Mean cover (%)					
**Alphonse Atoll, Seychelles (1997/98)**[1]	20-40	2001	17.9	2003	23.7	2	56.5-113	2.9	Pcp, Acr, Por ms	
East (5–15 m)	n.a.	—"—	18.2	—"—	22.2	—"—	n.a.	2.0	n.a.	
West (5–15 m)	—"—	—"—	17.6	—"—	25.2	—"—	—"—	3.8	—"—	
**Aldabra Atoll, Seychelles (2015/16)**[a]	30.2	2016	15.4	2019	22.0	3	71.4	2.2		Special Reserve (1981)
Lagoon (2 m)	32.3	—"—	21.0	—"—	30.0	—"—	92.9	3.0	Por br	—"—
Seaward west (5 m)	34.1	—"—	16.2	—"—	23.1	—"—	67.7	2.3	Mon	—"—
Seaward east (5 m)	24.1	—"—	9.1	—"—	12.9	—"—	53.6	1.3	Mon	—"—
**Kenya (1997/98)**[2,3]	31.3	1999	10.7	2002	17.7	3	56.4	2.3		
Malindi (lagoon, 2 m)	45.0	—"—	9.0	—"—	17.0	—"—	37.8	2.7	Por br, Pav	No-take MNP (mid 1970s)
Watamu (lagoon, 2 m)	38.0	—"—	10.0	—"—	13.0	—"—	34.2	1.0	Mon, Glx	No-take MNP (mid 1970s)
Mombasa (lagoon, 2 m)	43.0	—"—	14.0	—"—	25.0	—"—	58.1	3.7	Por br, Pav, Mon, Glx	No-take MNP (1991)
Vipingo (lagoon, 2 m)	20.0	—"—	11.0	—"—	23.0	—"—	115.0	4.0	Por br	
Kanamai (lagoon, 2 m)	24.0	—"—	17.0	—"—	23.0	—"—	95.8	2.0	—"—	
Ras Iwatine (lagoon, 2 m)	18.0	—"—	3.0	—"—	5.0	—"—	27.8	0.7	—"—	
**Lakshadweep Islands, India (1997/98)**[4,5]	60-90	2000	9.0	2003	19.4	3	21-32	3.5		
Agatti west (5–12 m)	n.a.	—"—	13.0	—"—	34.0	—"—	n.a.	7.0	Acr	
Kadmat west (5–12 m)	—"—	—"—	5.0	—"—	19.0	—"—	—"—	4.7	—"—	
Kavaratti west (5–12 m)	—"—	—"—	18.0	—"—	26.0	—"—	—"—	2.7	—"—	
Agatti east (5–12 m)	—"—	—"—	5.0	—"—	11.0	—"—	—"—	2.0	Not mentioned	
Kadmat east (5–12 m)	—"—	—"—	4.0	—"—	7.0	—"—	—"—	1.0	—"—	
**Maldives (1997/98)**[4]	n.a.	1998	2.9	2002	12.2	4	n.a.	2.3		
Bandos (reef flat, 1–2 m)	—"—	—"—	1.9	—"—	6.9	—"—	—"—	1.3	Pcp	
Fesdoo (reef flat, 1–2 m)	—"—	—"—	3.3	—"—	22.1	—"—	—"—	4.7	—"—	

(continued on next page)

Table S6 (continued)

Location (bleaching event)	Pre-bleaching mean cover (%)	First record post-bleaching		Last record post-bleaching		No. recovery years	Recovery (%)[b]	Annual rate of change (%)	Dominant recovery taxa	Level of protection (year of designation)
		Year	Mean cover (%)	Year	Mean cover (%)					
Gan (reef flat, 1–2 m)	—"—	—"—	4.0	—"—	12.9	—"—	—"—	2.2	—"—	
Villingili (reef flat, 1–2 m)	—"—	—"—	4.3	—"—	13.2	—"—	—"—	2.2	—"—	
Kooddoo (reef flat, 1–2 m)	—"—	—"—	1.0	—"—	6.0	—"—	—"—	1.3	—"—	
**Sri Lanka (1997/98)**[4]	72.6	1999	12.0	2004	32.8	5	40.2	4.2		
Bar Reef (0–3 m)	78.5	—"—	1.0	—"—	17.7	—"—	22.5	3.3	Pcp, Acr	Marine Sanctuary (1992)
Hikkaduwa (0–3 m)	47.2	—"—	7.0	—"—	10.1	—"—	21.4	0.6	Mon	
Weligama (0–3 m)	92.0	—"—	28.0	—"—	70.6	—"—	76.7	8.5	Acr	
**Thousand Islands, Indonesia (1982/83)**[6]	24.0	1983	2.5	1988	12.5	5	52.1	2.0		
South Pari (reef flat)	22.0	—"—	3.0	—"—	14.0	—"—	63.6	2.2	Not mentioned	
South Tikus (reef flat)	26.0	—"—	2.0	—"—	11.0	—"—	42.3	1.8	Mon	
**Palau (1997/98)**[7,8]	34.4	2002	19.5	2010	36.1	8	105.0	2.1		
Outer reef west (3 m)	28.3	—"—	13.0	—"—	30.0	—"—	105.9	2.1	Mon, Por, Mer	
Outer reef west (10 m)	31.0	—"—	25.0	—"—	50.0	—"—	161.3	3.1	—"—	
Outer reef east (3 m)	n.a.	—"—	11.0	—"—	35.0	—"—	n.a.	3.0	—"—	
Outer reef east (10 m)	—"—	—"—	18.0	—"—	35.0	—"—	—"—	2.1	—"—	
Patch reef (3 m)	—"—	—"—	7.5	—"—	25.0	—"—	—"—	2.2	Acr, Por	
Patch reef (10 m)	—"—	—"—	2.6	—"—	15.0	—"—	—"—	1.6	Acr, Por, Aga	
Inner reef (3 m)	43.5	—"—	45.0	—"—	59.0	—"—	135.6	1.8	Por	
Inner reef (10 m)	34.8	—"—	34.0	—"—	40.0	—"—	115.1	0.8	Por, Mer	
**Inner Islands, Seychelles (1997/98)**[9]	26.2	2005	11.4	2014	27.4	9	126.4	1.8		
Mahe E Patch (reef slope)	19.0	—"—	6.0	—"—	7.0	—"—	36.8	0.1	Acr	
Mahe NW Carbonate (reef slope)	39.0	—"—	10.0	—"—	38.0	—"—	97.4	3.1	—"—	No-take MNP (1979)[10]
Mahe W Carbonate (reef slope)	34.0	—"—	16.0	—"—	38.0	—"—	111.8	2.4	—"—	
Praslin NE Patch (reef slope)	26.0	—"—	5.0	—"—	9.0	—"—	34.6	0.4	Acr, Pcp	No-take MNP (1979)[10]
Ste Anne Granite (reef slope)	40.0	—"—	14.0	—"—	14.0	—"—	35.0	0.0	—"—	No-take MNP (1973)[10]

(continued on next page)

Table S6 (continued)

Location (bleaching event)	Pre-bleaching mean cover (%)	First record post-bleaching Year	First record post-bleaching Mean cover (%)	Last record post-bleaching Year	Last record post-bleaching Mean cover (%)	No recovery years	Recovery (%)[b]	Annual rate of change (%)	Dominant recovery taxa	Level of protection (year of designation)
Ste Anne Patch (reef slope)	55.0	—"—	8.0	—"—	43.0	—"—	78.2	3.9	Acr	No-take MNP (1973)[10]
Mahe NW Granite (reef slope)	11.0	—"—	10.0	—"—	36.0	—"—	327.3	2.9	Acr, Pcp	
Mahe NW Patch (reef slope)	18.0	—"—	15.0	—"—	25.0	—"—	138.9	1.1	—"—	
Mahe W Granite (reef slope)	19.0	—"—	16.0	—"—	35.0	—"—	184.2	2.1	—"—	
Mahe W Patch (reef slope)	19.0	—"—	29.0	—"—	31.0	—"—	163.2	0.2	—"—	
Praslin NE Granite (reef slope)	16.0	—"—	4.0	—"—	22.0	—"—	137.5	2.0	Not mentioned	No-take MNP (1979)[10]
Praslin SW Granite (reef slope)	18.0	—"—	4.0	—"—	31.0	—"—	172.2	3.0	Acr, Pcp	
**Chagos Archipelago, BIOT (1997/98)**[11]	39.8	2001	12.3	2012	40.8	11	102.5	2.6		No-take MPA (2010)[12]
Seaward slopes (5 m)	43.0	—"—	10.0	—"—	36.0	—"—	83.7	2.4	Iso, Acr	—"—
Seaward slopes (10 m)	44.0	—"—	11.0	—"—	50.0	—"—	113.6	3.5	Acr	—"—
Seaward slopes (15 m)	44.0	—"—	15.0	—"—	39.0	—"—	88.6	2.2	Not mentioned	—"—
Seaward slopes (25 m)	28.0	—"—	13.0	—"—	38.0	—"—	135.7	2.3	—"—	—"—
**Cocos Islands, Costa Rica (1982/83)**[13]	31.9	1987	3.2	2002	21.2	15	66.5	1.2		
Chatham (3-18 m)	29.1	—"—	2.9	—"—	16.7	—"—	57.4	0.9	Por	
Presidio (9-24 m)	34.7	—"—	3.5	—"—	25.7	—"—	74.1	1.5	Por, Pav	

[a] This study

[b] Percentage of ‘last record post-bleaching mean cover’ on ‘pre-bleaching mean cover’

Pcp = *Pocillopora*, Acp = *Acropora*, Por = *Porites*, Pav = *Pavona*, Mon = *Montipora*, Glx = *Galaxea*, Aga = Agaricidae, ms = massive, br = branching

n.a. = not available

BIOT = British Indian Ocean Territory

MNP = Marine National Park

MPA = Marine Protected Area

References to Table S6

1. Hagan, A. B. & Spencer, T. Reef resilience and change 1998-2007, Alphonse Atoll, Seychelles in *Proceedings of the 11th International Coral Reef Symposium* (eds. Riegl, B. & Dodge, R.) 388–392 (Nova Southeastern University National Coral Reef Institute, 2008).
2. McClanahan, T. R., Maina, J., Starger, C. J., Herron-Perez, P. & Dusek, E. Detriments to post-bleaching recovery of corals. *Coral Reefs* **24**, 230–246 (2005).
3. Baker, A. C., Glynn, P. W. & Riegl, B. Climate change and coral reef bleaching: an ecological assessment of long-term impacts, recovery trends and future outlook. *Estuar. Coast. Shelf Sci.* **80**, 435–471 (2008).
4. Rezai, H., Wilson, S. K., Claereboudt, M. & Riegl, B. Coral reef status of the ROPME sea area: Arabian/Persian Gulf, Gulf of Oman and Arabian Sea in *Status of Coral Reefs of the World: 2004* (ed. Wilkinson, C.) 155–170 (Global Coral Reef Monitoring Network and Australian Institute of Marine Science, 2004).
5. Arthur, R., Done, T. J., Marsh, H. & Harriott, V. Local processes strongly influence post-bleaching benthic recovery in the Lakshadweep Islands. *Coral Reefs* **25**, 427–440 (2006).
6. Brown, B. E. & Suharsono. Damage and recovery of coral reefs affected by El Niño related seawater warming in the Thousand Islands, Indonesia. *Coral Reefs* **8**, 163–170 (1990).
7. Gouezo, M. *et al.* Drivers of recovery and reassembly of coral reef communities. *Proc. R. Soc. B.* **286**, 20182908 (2019).
8. Bruno, J. F., Siddon, C., Witman, J., Colin, P. & Toscano, M. El Niño related coral bleaching in Palau, Western Caroline Islands. *Coral Reefs* **20**, 127–136 (2001).
9. Robinson, J. P. W., Wilson, S. K. & Graham, N. Abiotic and biotic controls on coral recovery 16 years after mass bleaching. *Coral Reefs* **38**, 1255–1265 (2019).
10. Jennings, S., Marshall, S. S., Cuet, P. & Naim, O. Chapter 13. The Seychelles in *Coral Reefs of the Indian Ocean. Their Ecology and Conservation* (eds. McClanahan, T. R., Sheppard, C. R. C. & Obura, D. O.) 383–410 (Oxford University Press, 2000).
11. Sheppard, C. & Sheppard, A. British Indian Ocean Territory (Chagos Archipelago) in *World Seas: An Environmental Evaluation. Volume II: The Indian Ocean to the Pacific* (ed. Sheppard, C.) 237–252 (Academic Press, 2018).
12. Sheppard, C. R. C. *et al.* Reefs and islands of the Chagos Archipelago, Indian Ocean: why it is the world's largest no-take marine protected area. *Aquat. Conserv.* **22**, 232–261 (2012).
13. Guzman, H. M. & Cortés, J. Reef recovery 20 years after the 1982-1983 El Niño massive mortality. *Mar. Biol.* **151**, 401–411 (2007).

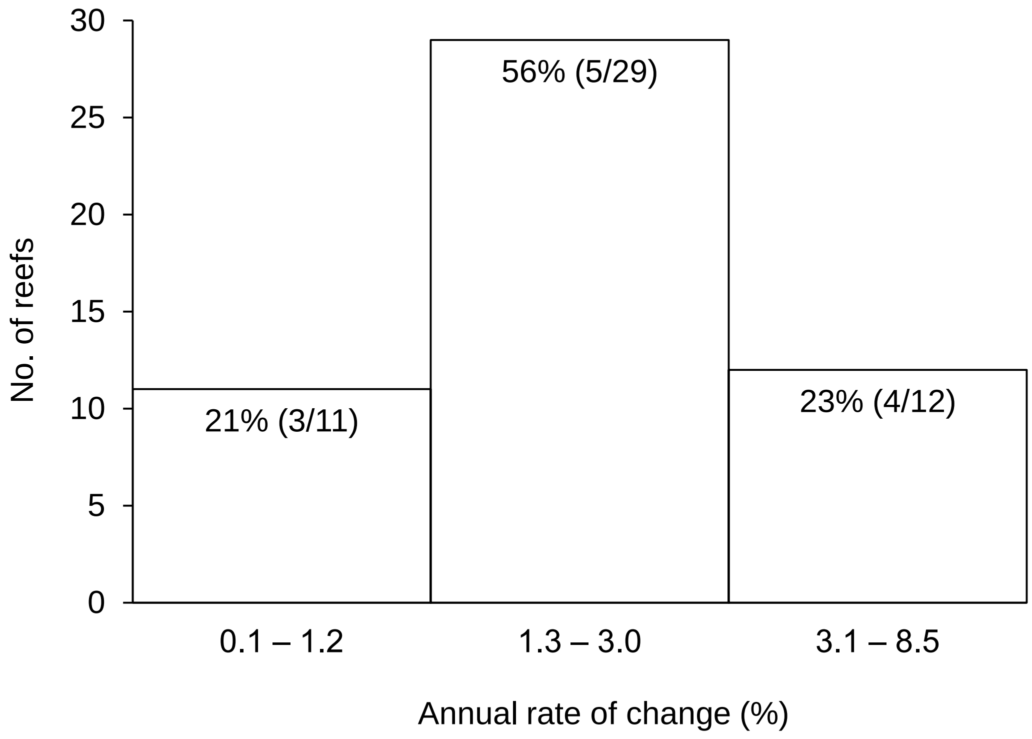

Figure S4: Histogram of the annual rates of change of the 52 reefs displayed in Table S6. Number of reefs (percentage on total shown in bins) with annual rates of change that fall within the same range as Aldabra's reefs (1.3–3.0%) as well as below and above. Values in brackets indicate the number of protected areas on the total number of reefs within the bin (reefs of the Chagos Archipelago not counted as protected area here as designation happened after the majority of recovery years).

Table S7: Cover of fleshy macroalgae between 2014 and 2019. Cover (mean ± SE) based on two (2014) and three (2016–2019) transect sections per site. At Site 5 (15 m) surveyed in December 2018, cover was entirely due to a *Caulerpa racemosa* bloom which had subsided by February 2019 (pers. obs. AK, February 2019).

Depth	Location	2014	2016	2017	2018	2019
2 m	**Lagoon**	**0.37 ± 0.19**	**0.06 ± 0.03**	**0.27 ± 0.12**	**0.46 ± 0.14**	**0.30 ± 0.11**
	Site 9	0.00 ± 0.00	0.12 ± 0.06	0.58 ± 0.27	0.32 ± 0.24	0.54 ± 0.20
	Site 10	0.51 ± 0.51	0.00 ± 0.00	0.22 ± 0.15	0.56 ± 0.21	0.30 ± 0.22
	Site 11	0.61 ± 0.23	0.06 ± 0.06	0.00 ± 0.00	0.50 ± 0.35	0.05 ± 0.05
5 m	**West**	**0.05 ± 0.02**	**0.02 ± 0.02**	**0.02 ± 0.02**	**0.18 ± 0.07**	**0.07 ± 0.03**
	Site 1	0.09 ± 0.09	0.06 ± 0.06	0.00 ± 0.00	0.00 ± 0.00	0.17 ± 0.10
	Site 2	0.08 ± 0.08	0.00 ± 0.00	0.00 ± 0.00	0.05 ± 0.05	0.00 ± 0.00
	Site 6	0.00 ± 0.00	0.00 ± 0.00	0.00 ± 0.00	0.31 ± 0.09	0.00 ± 0.00
	Site 7	0.08 ± 0.08	0.00 ± 0.00	0.11 ± 0.06	0.42 ± 0.27	0.11 ± 0.11
	Site 8	0.00 ± 0.00	0.06 ± 0.06	0.00 ± 0.00	0.11 ± 0.11	0.06 ± 0.06
	**East**	**0.01 ± 0.01**	**0.05 ± 0.05**	**0.00 ± 0.00**	**0.12 ± 0.12**	**0.13 ± 0.11**
	Site 3	0.00 ± 0.00	0.00 ± 0.00	0.00 ± 0.00	0.00 ± 0.00	0.06 ± 0.06
	Site 4	0.05 ± 0.05	0.19 ± 0.19	0.00 ± 0.00	0.00 ± 0.00	0.00 ± 0.00
	Site 5	0.00 ± 0.00	0.00 ± 0.00	0.00 ± 0.00	0.48 ± 0.48	0.45 ± 0.45
	Site 12	0.00 ± 0.00	0.00 ± 0.00	0.00 ± 0.00	0.00 ± 0.00	0.00 ± 0.00
15 m	**West**	**0.01 ± 0.01**	**0.55 ± 0.14**	**0.36 ± 0.10**	**0.40 ± 0.17**	**0.25 ± 0.05**
	Site 1	0.00 ± 0.00	0.61 ± 0.42	0.17 ± 0.08	0.06 ± 0.05	0.21 ± 0.12
	Site 2	0.00 ± 0.00	0.50 ± 0.27	0.17 ± 0.16	0.28 ± 0.11	0.01 ± 0.00
	Site 6	0.04 ± 0.04	0.66 ± 0.35	0.51 ± 0.29	1.30 ± 0.69	0.29 ± 0.11
	Site 7	0.00 ± 0.00	0.92 ± 0.42	0.71 ± 0.27	0.12 ± 0.05	0.41 ± 0.06
	Site 8	0.00 ± 0.00	0.08 ± 0.07	0.22 ± 0.14	0.22 ± 0.11	0.30 ± 0.16
	**East**	**0.00 ± 0.00**	**0.15 ± 0.06**	**1.53 ± 1.49**	**9.75 ± 5.70**	**0.02 ± 0.01**
	Site 3	0.00 ± 0.00	0.07 ± 0.06	0.01 ± 0.00	0.06 ± 0.05	0.07 ± 0.06
	Site 4	0.00 ± 0.00	0.25 ± 0.16	0.12 ± 0.06	0.01 ± 0.00	0.01 ± 0.00
	Site 5	0.00 ± 0.00	0.27 ± 0.18	5.97 ± 5.96	38.93 ± 12.11	0.01 ± 0.00
	Site 12	0.00 ± 0.00	0.01 ± 0.00	0.01 ± 0.00	0.01 ± 0.00	0.01 ± 0.00

Equation 1 and 2

To further assess reef recovery, we calculated the annual rate of change in absolute hard coral cover increase (C_{RC}) following Coté et al[14]:

$$C_{RC} = \frac{C_{END} - C_{START}}{Y} \quad (1)$$

where C_{START} is the hard coral cover immediately post-bleaching (2016), C_{END} is the most recent record of hard coral cover (2019) and Y is the number of years between the two values. Based on the above, we obtained an estimate of years remaining (from 2019) until hard coral cover reaches pre-bleaching levels (Y_R):

$$Y_R = \frac{C_{PRE} - C_{END}}{C_{RC}} \quad (2)$$

where C_{PRE} is the pre-bleaching hard coral cover (2014).

Reference:

14. Coté, I. M., Gardner, T. A., Hutchinson, D. J. & Watkinson, A. R. New approaches to estimating recent ecological change on coral reefs in *Coral Reef Conservation* (eds. Coté, I. M. & Reynolds, J. R.) 293–313 (Cambridge University Press, 2006).
